# Malaria Control and Elimination in Sri Lanka: Documenting Progress and Success Factors in a Conflict Setting

**DOI:** 10.1371/journal.pone.0043162

**Published:** 2012-08-29

**Authors:** Rabindra R. Abeyasinghe, Gawrie N. L. Galappaththy, Cara Smith Gueye, James G. Kahn, Richard G. A. Feachem

**Affiliations:** 1 Country Office, World Health Organization, Port Moresby, National Capital District, Papua New Guinea; 2 Anti-Malaria Campaign, Ministry of Health, Colombo, Western Province, Sri Lanka; 3 Global Health Group, University of California San Francisco, San Francisco, California, United States of America; 4 Institute for Health Policy Studies, University of California San Francisco, San Francisco, California, United States of America; Tulane University School of Public Health and Tropical Medicine, United States of America

## Abstract

**Background:**

Sri Lanka has a long history of malaria control, and over the past decade has had dramatic declines in cases amid a national conflict. A case study of Sri Lanka's malaria programme was conducted to characterize the programme and explain recent progress.

**Methods:**

The case study employed qualitative and quantitative methods. Data were collected from published and grey literature, district-level and national records, and thirty-three key informant interviews. Expenditures in two districts for two years – 2004 and 2009 – were compiled.

**Findings:**

Malaria incidence in Sri Lanka has declined by 99.9% since 1999. During this time, there were increases in the proportion of malaria infections due to *Plasmodium vivax*, and the proportion of infections occurring in adult males. Indoor residual spraying and distribution of long-lasting insecticide-treated nets have likely contributed to the low transmission. Entomological surveillance was maintained. A strong passive case detection system captures infections and active case detection was introduced. When comparing conflict and non-conflict districts, vector control and surveillance measures were maintained in conflict areas, often with higher coverage reported in conflict districts. One of two districts in the study reported a 48% decline in malaria programme expenditure per person at risk from 2004 to 2009. The other district had stable malaria spending.

**Conclusions/Significance:**

Malaria is now at low levels in Sri Lanka – 124 indigenous cases were found in 2011. The majority of infections occur in adult males and are due to *P. vivax*. Evidence-driven policy and an ability to adapt to new circumstances contributed to this decline. Malaria interventions were maintained in the conflict districts despite an ongoing war. Sri Lanka has set a goal of eliminating malaria by the end of 2014. Early identification and treatment of infections, especially imported ones, together with effective surveillance and response, will be critical to achieving this goal.

## Introduction

**Figure 1:**
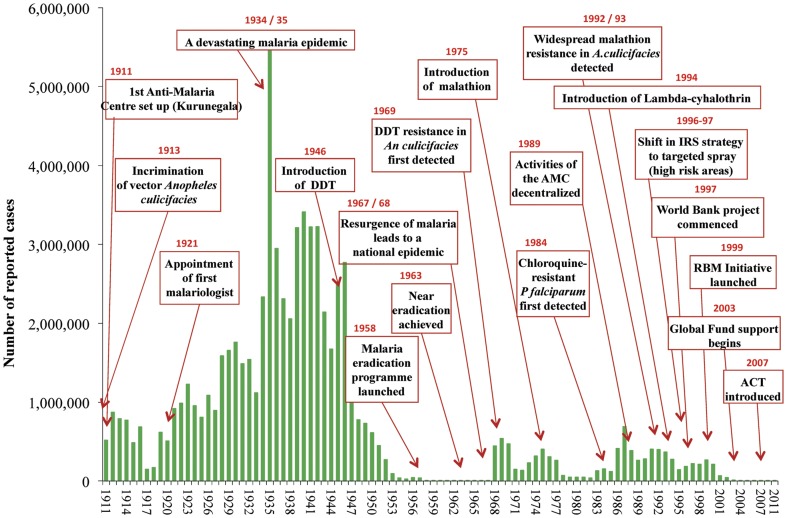
Timeline of reported cases and major events in Sri Lanka, 1911–2011.

In AD 300, the former capital city of Sri Lanka, Anuradhapura, was devastated by a “pestilence” that was likely malaria. From AD 1300 onwards, indigenous medical literature describes a fever that echoes the “chill, rigor, gooseskin, and headache” of malaria [1]. In 1908 the first spleen survey was carried out and by 1921, the island, then known as Ceylon, appointed its first malariologist [1], [2]. Epidemics occurred every three to five years, a major one occurring from 1934–1935 that led to an estimated 5.5 million cases (see Figure 1) [1]. In 1945, Sri Lanka became the first country in the region to develop a scheme for indoor residual spraying (IRS) using DDT and established its first mobile spray unit. IRS was quickly expanded to all malarious areas [1]. At the same time, “vigilance units” conducted parasitological and entomological surveillance, including active surveillance [3], [4]. In 1954 as a result of declining cases, IRS was reduced but then was quickly redeployed in response to rising malaria [1]. In 1958, Sri Lanka joined the Global Malaria Eradication Programme [5].

A massive decline in incidence occurred in Sri Lanka, from 91,990 cases in 1953 to 17 cases in 1963 [3], [6], [7]. Then, as was the case for many other countries [8], [9], IRS was scaled down, surveillance and control activities were relaxed, and financial support reduced [1], [10]–[12]. In combination with reduced rainfall in the wet zone [13], these actions led to a massive resurgence, with an estimated 1.5 million cases during the two-year period 1967–1968 [14]. IRS was scaled back up the next year, but the damage had already been done. Major epidemics have since occurred in Sri Lanka in the 1980s and early 1990s [15].

Since 1999, Sri Lanka has seen a dramatic decline in malaria once again. This success is notable given the major operational challenges posed by nearly 30 years of civil war between the Liberation Tigers of the Tamil Eelam (LTTE) and the Sri Lankan Government (1983 to 2009). There are many examples throughout the world of the negative consequences of conflict on the function of malaria control programmes [16]–[19]. However, from 1999 onwards, Sri Lanka achieved major reductions in incidence and may now be considered a controlled low-endemic country [20], [21]. A century (1911–2011) of malaria incidence and relevant events is summarized in Figure 1. Sri Lanka aims to interrupt indigenous malaria transmission, or eliminate [8], *Plasmodium falciparum* by the end of 2012 and *Plasmodium vivax* by the end of 2014 (See Table 1) [6], [22]–[24]

**Table 1:** Malaria transmission factors in Sri Lanka.

Proportion of cases due to *Plasmodium vivax*	90.3% (2011)
Populations considered to be most at risk	Security forces personnel, gem miners, mobile populations
Vectors	Principal vector is *Anopheles culicifacies*, species E; *An. subpictus* is considered a minor vector
Malaria geography and seasonality	Malaria transmission has historically occurred north, east and southeast; Malaria transmission typically peaks from December to March, with a smaller peak from June to October.

While the history of Sri Lanka's battle with malaria is interesting, this case study focuses on the last 15 years of the successful malaria programme of Sri Lanka, describing the malaria epidemiology and the important factors that have led to the sustained decline in malaria. The aim is to provide a description of the country's experience and the lessons learned as it has moved toward elimination, from which other countries may benefit. Socio-economic and political enabling and challenging factors are described, with a particular focus on the civil conflict and whether surveillance and vector control efforts were maintained in conflict areas. Expenditures on malaria control were measured in two districts, to see how the cost of malaria control per capita at risk changed as the country moved from a highly endemic period to one of controlled, low-endemic malaria.

## Methods

### Geography, population and climate

Sri Lanka is an island in the Indian Ocean, to the southeast of India. This lower-middle income country has a population of 20.2 million [25]. Sri Lanka has 25 districts, of which six are considered to be at very low to no risk for malaria. Cases reported in these districts are likely to have originated in other districts. Malaria transmission is seasonal, typically peaking at the end of the northeast monsoon season (December to March), with a smaller peak after the southwest monsoon (June to October). There are three climatic zones: the southwest forms a wet zone; the northwest and western mountain slopes form an intermediate wet zone; and a dry zone encompasses the north, east and southeast [13], [26]. Malaria transmission has been considered endemic in the dry zone and epidemic-prone in the intermediate zone. The wet zone is historically an area of limited vector breeding as a result of continual precipitation which flushes out the rivers and streams.

### Data sources

#### Desk research

A review of published and unpublished literature was conducted before the start of field work, then again during and after in-country data collection. Documents collected before field work informed the key informant interviews and quantitative data collection. Documents collected during and after field work were used in the analysis, as described below. A search was conducted using Google, Google Scholar, Pubmed, World Health Organization Library (WHOLIS) [27], World Health Organization (WHO) Office of the South-East Asia Region [28], and the Global Fund to Fight AIDS, Tuberculosis and Malaria (Global Fund) website using the search terms “Sri Lanka” AND “malaria” AND “case management,” OR “diagnosis,” OR “treatment,” OR “prevention,” OR “surveillance,” OR “elimination,” OR “conflict,” OR “*Plasmodium vivax* OR *Plasmodium falciparum*,” OR “G6PD.” References were also identified by cross-referencing bibliographies of relevant publications. The review included grey literature obtained from the Anti-Malaria Campaign Directorate and offices of the Regional Malaria Officers (see Programme structure section, below) during the field data collection period, such as annual reports, administrative reports and plans, and grant reports. Inclusion criteria included any articles that included the above key words and were in English. The exclusion criteria were not including the key words and articles written in languages other than English.

#### Quantitative data

Data on malaria testing and incidence were pulled from routine health facility surveillance records of the Anti-Malaria Campaign (AMC) Directorate and Regional Malaria Officers (RMOs) for 1995 to 2011. The AMC Directorate provided district-level annual estimates of population at risk, indoor residual spraying (IRS) activities, and distribution of insecticide-treated nets (ITNs) and long-lasting insecticide-treated net (LLINs).

Most expenditure records were gathered from hard copy and electronic files from the offices of the Regional Directors of Health Services (RDHSs) and Regional Malaria Officers. Commodities (e.g., LLIN procurement) were identified through record review and interviews at the AMC Directorate in Colombo. Costs were reported in Sri Lankan Rupees (LKR) and U.S. Dollars (USD). The costing analysis does not include expenditures or contributions to the malaria programme from non-public sector entities, such as by households, non-governmental organizations (NGOs), or Global Fund support through organizations outside of the public sector.

Expenditure data were gathered from two of the largest malarious districts, Anuradhapura and Kurunegala. The districts were chosen based on their differing characteristics, level of experience of malaria programme managers, and safety of travel to the districts at the time of this study [29]. Because of the difficulty in assembling costing data, two years were chosen - 2004 and 2009 - to represent different phases of the district malaria programme as identified by epidemiological data and programmatic shifts; from endemic or epidemic malaria (2004) to controlled low-endemic malaria (2009). Since malaria programme staff also work on other vector-borne diseases, the key informant interviews and a review of job descriptions were used to determine the proportion of time spent on malaria.

#### Key informant interviews

Thirty-three in-person semi-structured key informant interviews, using an interview guide, were conducted at the AMC Directorate office and in the RMOs and Medical Officer of Health Area (MOH Areas) Offices of the districts of Ampara, Anuradhapura and Kurunegala. Seven interviews were conducted at the AMC Directorate, including managers, entomological and parasitological laboratory staff, and accountants. Nineteen total interviews were conducted at RMOs in the three sample districts, ranging from programme managers to entomologists, technical support staff to IRS spraymen to drivers. Six interviews were conducted at Medical Officer of Health Areas, ranging from managers to spraymen. One interview was conducted at a Regional Director of Health Services office with an accountant. No interviews were conducted outside of the public sector. A purposeful sampling method was used to identify knowledgeable subjects for the interviews. The AMC Directorate programme manager identified five RMOs with extensive experience, who in turn suggested other staff members for interviews based on the subjects in the interview guide and the gaps in data. Verbal consent was obtained before the interviews.

#### Political, environmental and socio-economic data

**Figure 2:**
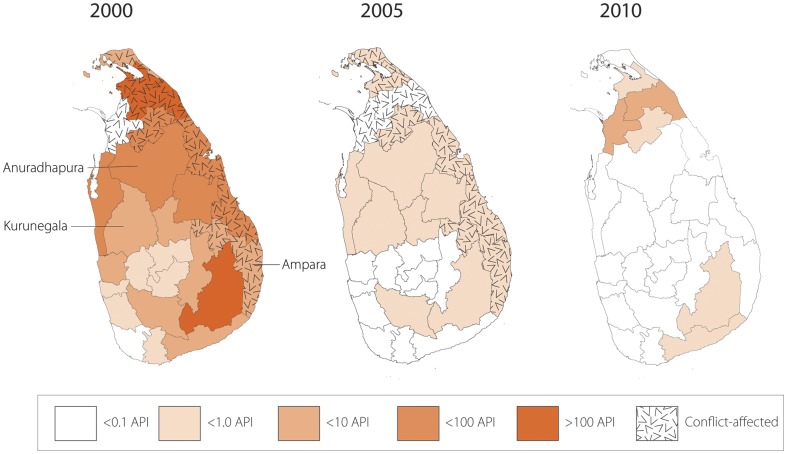
Map of Annual Parasite Incidence (API) (confirmed infections/1,000 population at risk) by district, 2000, 2005, and 2010. API per 1,000/population at risk. The costing analysis was conducted in Anuradhapura and Kurunegala districts. Key informant interviews were conducted with representatives from Ampara, Anuradhapura, and Kurunegala districts. The Malaria Atlas Project (MAP) and the Sri Lanka Ministry of Health provided the base district-level map of Sri Lanka. MAP is committed to disseminating information on malaria risk, in partnership with malaria endemic countries, to guide malaria control and elimination globally.

There was a civil conflict between government forces and the LTTE from 1983 to 2009. While government forces reclaimed two eastern districts in 2008, the remaining conflict districts shown in Figure 2 remained under LTTE control until the war was declared over in May 2009. A conflict variable was created, whereby districts were considered to be “non-conflict” if they were under government control without indication of active conflict. The sources of data for this variable were the Sri Lanka Ministry of Defense conflict maps and the LexisNexis Academic database, with a search of terms “Sri Lanka,” AND “conflict” OR “war” OR “LTTE” [30]. If there was a conflict between these two sources, the Ministry of Defense reports were used as the deciding factor.

Population health, health expenditure, and economic indicators were accessed from the World Bank [25].

### Analysis

The quantitative and qualitative data were reviewed to identify factors that contributed to the decline in malaria in Sri Lanka, including an estimate of the coverage of vector control and surveillance across conflict and non-conflict districts. Information from the literature found in the desk review collected before the commencement of field work was used to formulate the quantitative and qualitative data collection tools, such as the interview guides and Microsoft Excel spreadsheets for surveillance data. These documents, in addition to new sources of grey literature accessed during and after in-country data collection, served to identify the major changes in malaria control strategies and interventions. These preliminary findings were compared to the qualitative and quantitative data collected in the field. In later stages of analysis, these documents served to fill gaps in data or were used to confirm or raise questions about conclusions that were developed from the interviews and quantitative data.

Annual, district-level data on malaria incidence, surveillance and vector control activities were plotted in Microsoft Excel. Major malaria indicators and coverage estimates were calculated and trends observed over time. Major political, socio-economic and environmental trends, with a focus on conflict districts, were reviewed. All of these trends were then compared with each other through data triangulation, which is defined as the review, synthesis and interpretation of data from multiple sources. A wide variety of data sources may be examined through data triangulation, from programme data to biological or behavioral data, with a goal of informing public health decision-making [31]. If differences emerged across data sources in the case study, the key informant interviews were considered the primary source of information.

Costs were categorized into personnel, travel, equipment, consumables, and services. They were also grouped by intervention category: prevention, diagnosis, treatment and prophylaxis, surveillance and response, information education and communication (IEC), and programme management. Costs of equipment were amortized using straight-line depreciation. All costs were converted into 2009 U.S. dollars using in-country deflators and 2009 representative country exchange rates [32], [33]. As district-level costs included contributions by only the RDHS or district-level budget and Global Fund support, funding provided by the national Ministry of Health for malaria (e.g., some personnel) was estimated and allocated proportionally across each intervention for the two districts. The national budget report was used to calculate funding provided by the Ministry of Health. The 2008 national budget report was used to calculate funds for malaria for 2009. National costs for 2004 and 2008 were assigned to districts in proportion to total district spending on malaria control.

### Ethical considerations

Ethical clearance, with the conduct of verbal consent procedures, was obtained from the Committee on Human Research at the University of California, San Francisco. An approved verbal consent guide was read to participants and their consent was noted. The Ministry of Health in Sri Lanka approved the conduct of the case study. Informed verbal consent was obtained for all key informant interviews and data from the Ministry of Health and Anti-Malaria Campaign were analyzed in aggregate, without information that might identify individuals. The case study was considered to be a low-risk behavioral study thus verbal consent was deemed appropriate.

## Results

### Desk research

The desk research identified 112 publications related to malaria control and elimination in Sri Lanka. Roughly a quarter (26) of these publications are studies on vector control in Sri Lanka. There were 72 grey documents identified and reviewed, 56 of which were reports from the Anti-Malaria Campaign, the Sri Lanka Ministry of Health, or reports written by consultants about a project implemented by either organization. There were 16 documents reviewed from the World Health Organization. A list of these documents is available in Appendix S1.

### Programme structure

**Figure 3:**
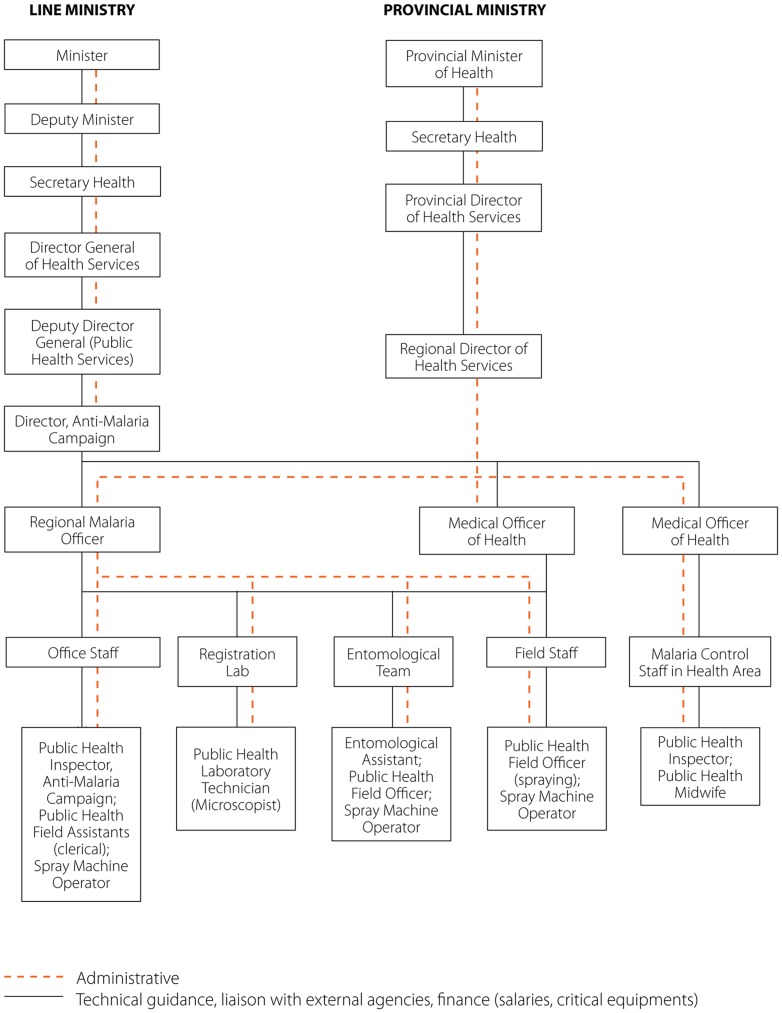
Organizational diagram of the Sri Lanka Anti-Malaria Campaign.

The Anti-Malaria Campaign (AMC) Directorate in Colombo guides and coordinates all malaria control activities (see Figure 3). Under the purview of the AMC is formulation of national malaria control policy, monitoring national malaria trends, technical guidance to subnational malaria control programmes, inter-district coordination, and coordination of training and research activities. Entomological and parasitological surveillance is also undertaken by the AMC. Decentralization in 1989 shifted the administration of malaria control activities to the districts. Health services are managed by the Regional Director of Health Services (RDHS) and responsibility for malaria control activities rests with the Regional Malaria Officer (RMO) in each district. RMOs work jointly with the Medical Officers of Health (MOHs), whose offices provide varying levels of support for vector control activities.

### Challenging and enabling factors

#### Conflict

Sri Lanka's long-running civil conflict affected the whole country but was concentrated in an estimated eight districts [34]. A ceasefire agreement was declared in 2002, and there was a decline in civilian casualties around this time: a decline in “battle-related deaths” was reported from 4,000 (2000) to 1,000 (2001), then down to zero in 2002 and 2004 [25]. The ceasefire also linked the Jaffna Region in the north to the rest of Sri Lanka through regular commercial passenger flights and the reopening of route A9, a major artery for transportation between Jaffna and Colombo [34], [35]. As a result, delivery of supplies to the northern areas may have increased. However, for the purposes of this case study, the number of districts considered to have conflict during these years remained at eight because reports indicate that the ceasefire was not respected by both sides at all times and sporadic fighting continued [34].

The ceasefire officially ended in 2006 when violence resumed in the northeast [34]. The same eight districts are considered to have had active conflict from 2005 to 2007, decreasing to six in 2008 and to four in 2009. Over this period deaths related to the conflict rose, peaking in 2008 (11,144) [25]. By May 2009 the war was declared over and by December route A9 was again open.

#### Socio-economics, health and environmental factors

National gross domestic product (GDP) per capita increased from $715 (current USD) in 1995 to $2,375 in 2010 [25]. Total health expenditure per capita (current USD) also rose from $26 in 1995 to $84 in 2009. The adult literacy rate in Sri Lanka was estimated to be 91% both in 2001 and in 2006. 76.6% of the population was reported to have access to electricity across the country in 2009 [25].

Historically, transmission of malaria has increased in Sri Lanka when pooling occurs in rivers and streams, which is conducive for the breeding of the primary vector, *Anopheles culicifacies.* Transmission increases with monsoon rain events in the dry zone. Transmission may also increase when the monsoon is weak or does not occur in the intermediate zone [7]. The literature did not report any major droughts, flooding, or shifts in vector breeding during the period 1995–2011. However, the World Bank Health Services Project reported that a drought in 2001 may have contributed to project-area declines in malaria incidence [36]. Studies have had reasonable success in linking rainfall to malaria incidence, when using a two to three month period for forecasting [37].

The tsunami of December 2004 led to a massive loss of life and displacement of 860,000 people. Hospitals and administrative buildings were destroyed [38]. However, reports indicate that surveillance and prevention activities for malaria were maintained by local health authorities and NGOs and no malaria epidemic accompanied the tsunami (2004–2005) [39].

### Epidemiology of malaria and vectors

**Figure 4:**
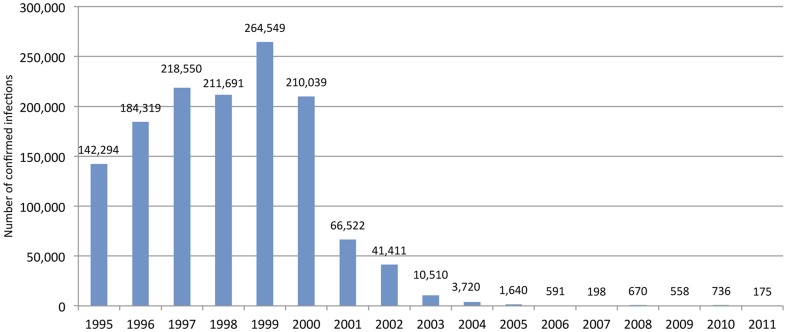
Total confirmed infections from Active and Passive Case Detection, Sri Lanka, 1995 to 2011.

From 1995 to 1999, the number of malaria infections confirmed by microscopy or rapid diagnostic tests (RDTs), or confirmed infections, rose from 142,294 to 264,549 (Figure 4). Then, beginning in 2000, cases began to decline. From 1999 to 2007, cases were reduced from 264,549 to 198 (99.9%). There was a small uptick in total cases, combined indigenous and imported, to 670 in 2008 through to 2010 (736). In 2011, 175 cases were confirmed, of which 124 were indigenous, meaning that the infection originated in Sri Lanka.

Malaria-related mortality in Sri Lanka has similarly declined, from a peak of 115 deaths in 1998 to zero indigenous deaths each year since 2008. In years 2009 and 2011 there was one death, each an imported case from Nigeria.

Annual parasite incidence (API), or the number of confirmed infections of all Plasmodium species divided by the estimated population at risk, was 11.9 per 1,000 in 1995, reached a peak of 22.1 in 1999, then declined to less than 1 by 2004 (0.9). In 2010 the estimated API of indigenous cases was 0.1. The slide positivity rate (SPR), or the proportion of slides found positive for indigenous cases for any Plasmodium parasites among the slides collected [40], was 13.0% in 1995, peaked in 1999 at 16.7%, then declined starting in 2000 (11.8%) to 0.2% in 2005. In 2010 the SPR was 0.1%. Studies employing polymerase chain reaction (PCR) assay have found no evidence of sub-microscopic parasitaemia in previously endemic portions of the country [41], [42].

**Figure 5:**
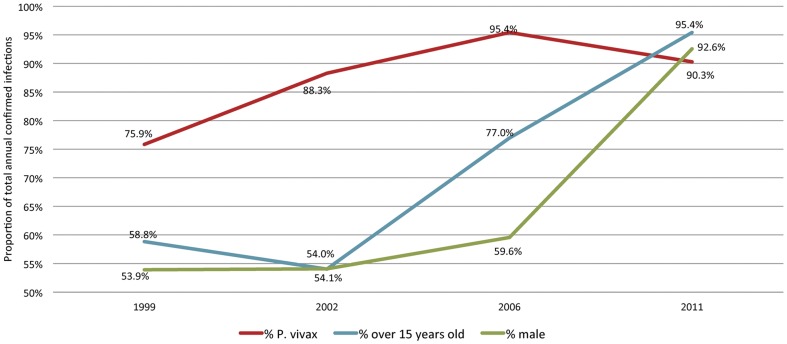
Annual percentage of confirmed infections for 1999, 2002, 2006 and 2011. All percentages represent total cases, indigenous and imported cases combined.

As national malaria morbidity declined, the profile of all people infected with malaria, indigenous and imported cases combined, became mostly adult males (ages 15–49) with *P. vivax* infections, as opposed to *P. falciparum* infections. The proportion of all confirmed cases occurring in persons over the age of 15 was 58.8% in 1999, 77.0% in 2006, and 95.4% in 2011 (Figure 5). In 1999, 53.9% of all infections were in males. By 2006 this grew to 59.6% and in 2011 reached 92.6% of cases. The proportion of infections due to *P. vivax* in all cases, including indigenous and imported, grew from 75.9% in 1999 to 95.4% in 2006 before leveling off at 90.3% in 2011. One *P. ovale* infection was diagnosed in 2005, then one *P. malariae* in 2008, which was acquired outside of Sri Lanka.

Male gem miners and male military personnel became a major risk group for malaria infection. Other at-risk groups are considered to be people living along rivers and streams with high vector density and mobile populations, such as chena (slash and burn) cultivators.

#### Conflict zone

Non-conflict districts (17 districts) had a similar API (33.0) to the eight conflict districts (29.9) in 1995, but by 2000 conflict districts accounted for the majority of infections (71.5 average API in conflict and 35.1 in non-conflict districts). In 2005, when national incidence was much lower, API across both areas was the same (0.4). Figure 2 shows the spatial distribution of API across districts during 2000, 2005, and 2010.

The SPR was higher in conflict districts throughout most of the study period. In 1995, conflict districts had an SPR of 17.0% compared to 11.7% for non-conflict districts. As seen with API, by 2005 the SPR was the same across both regions (0.2%) and has not changed in recent years.

#### Vectors

The principal vector in Sri Lanka is of the *Anopheles culicifacies* species E of the complex which has sibling species A, B, C, D, and E [22], [23]. Species B is considered a poor vector in Sri Lanka [23]. The species complex is largely found in stream habitat [43]. Species E can be found in a wider range of habitat, mainly in river margins in the rock and sand pools, agricultural sites, and in wells and irrigation channels [23]. Throughout its history, Sri Lanka has had major irrigation projects, funneling its rivers into dams, reservoirs and tunnels [7]. The vector is considered both endo- and exophagic (outdoor and indoor feeding behavior), primarily endophilic (indoor resting), an “intensely domestic species,” and has a dusk to night biting time [2], [44]. *An. culicifacies* is considered to be zoophilic in nature, except Species E [23]. In southeastern Sri Lanka in the 1990s, which was a period of higher endemicity, the entomological inoculation rate (EIR) of the ten Anopheles species studied was estimated at 0.0029 infectious bites per person per night for *P. vivax* and 0.0109 for *P. falciparum* [45]. More recent EIR estimates are not available as the number of vectors have been extremely low and finding sporozoite-infected mosquitoes even more challenging.

Another important vector on the island is *An. subpictus*, of which two sibling species are present in Sri Lanka, Species A and B. Species A is associated with inland areas and Species B with coastal areas [24]. *An. subpictus* is found in coastal and brackish water, and pools and rice fields [44]. This vector is also considered to be zoophilic and is both endo- and exophagic [44]. It is endophilic in nature and bites at dusk or night. A study in the eastern part of Sri Lanka in 1989 and 1990 estimated an EIR for *An. subpictus* ranged from 0.00006 to 0.007 infectious bites per night for *P. vivax* and from 0.0002 to 0.005 for *P. falciparum* [46].

### Parasitological surveillance

Two main surveillance measures are used in Sri Lanka – passive and active case detection. “Activated passive case detection,” or APCD, is a form of passive case detection used in Sri Lanka comprised of dedicated malaria-only screening facilities in public health facilities. APCD capacity increased in the late 1990s by a near doubling of the number of microscopists in district hospitals. Outside of APCD facilities, health facilities conduct passive case detection without malaria-specific screening centers. All of these facilities rely mostly on microscopy. RDTs were distributed starting in 2001, but are only for emergencies, such as in the months following the tsunami. 45,000 RDTs were procured in 2004 with Global Fund funding. If all RDTs were used, this figure would represent only 4% of the 1.2 million malaria tests conducted that year.

Limited clinical diagnosis still occurs but is discouraged by the Directorate, and clinicians are instructed to take a blood film two weeks later if possible. Quality control of microscopically-confirmed diagnosis occurs at the regional and national laboratories: regional laboratories perform quality control of all positive and negative blood smears while the national reference laboratory carries out quality control on all positive tests, including RDT-positive, and 10% of negative smears.

Active case detection (ACD) was introduced in 1997. Over the years, mobile malaria clinics have targeted mobile populations resulting from the conflict and remote, inaccessible populations in all areas. Today ACD is also part of the reactive case investigation procedures (focal screening). The aim is to detect asymptomatic and symptomatic parasite carriers, including relapsing *P. vivax* cases, who may contribute to post-monsoon epidemics. RDTs are occasionally used for these clinics, but the majority of tests are conducted by microscopy. The World Bank International Development Association (IDA) supported initial ACD activities and the Global Fund increased support for them starting in 2003.

The annual blood examination rate (ABER), or the number of blood slides collected out of the total national population, was 6.1% in 1995, 9.4% in 2000, and declined to 5.0% in 2005 with little change through 2010 (4.8%).

In all years, the majority of confirmed cases nationwide were identified through APCD. In 1995, 89.8% of cases were identified through this method, with no significant change in 2000 (89.4%). APCD identified 94.0% of all cases in 2005. ACD accounted for only a small percentage of positive cases (0.9%) in 1997 and 2000 (1.1%), rising to 13.1% in 2007. Passive case detection and other blood surveys found the remaining confirmed infections.

As the number of cases declined after 1999, district-level staff had more time to dedicate to case investigation. In 2009, the AMC Directorate developed standard operating procedures (SOPs) for every confirmed or suspected infection, which include follow-up of confirmed infections for 28 days post-treatment together with case investigation procedures and additional measures, such as household malaria screening, entomological surveillance within 24 hours, and focal IRS in a one-kilometer radius. Also in 2009, the programme instituted case investigation reviews, where each case and the follow up measures taken are reviewed in detail by AMC Directorate staff and Regional Malaria Officers, at a meeting in the capital. The information gathered in the case investigation and in the case reviews is used to detect any deviations in vector behaviour (see Entomological Surveillance section, below) as well as to monitor clinical manifestations and parasite clearance time. The results of these investigations, in combination with mapping with geographical information systems (GIS) technology, which commenced three years ago, are used for the purpose of epidemic forecasting, the cornerstone of the national malaria elimination strategy. The programme continually seeks to strengthen surveillance in order to quickly detect epidemics.

In 2008, the AMC Directorate introduced individual case reporting to the AMC and a year later a policy of 24-hour case reporting was implemented. Regional Malaria Officers report cases by email, by phone or through the hotline maintained by the AMC Directorate. An elimination surveillance database was developed to house this information and for rapid analysis. There is a national health information system and there is a national requirement to report malaria cases to this system. However, the AMC uses a separate, web-based system that enables the programme to conduct 24-hour reporting. The AMC expects to integrate the malaria reporting system with the national system and with other diseases once malaria is eliminated from the country.

Monitoring and evaluation is an important aspect of the surveillance system and the entire programme, and the systems in place have been greatly strengthened through implementation of the Global Fund grants. A framework and plan for monitoring and elimination was developed in 2010, based on the framework put forward in 2009 by the Roll Back Malaria (RBM) – Malaria Evaluation Reference Group (MERG) [47]. Indicators for disease surveillance and management as well as vector surveillance and control are included in the plan. Monitoring and evaluation of malaria activities is coordinated by the Regional Malaria Officers, at the district level, and at the national level by the AMC Directorate. Data is collected at the periphery at the smallest administrative level (Grama Niladari Division), with processing carried out by the RMOs and Medical Officer of Health Area staff [48]. Data is used either immediately for corrective action or is processed upwards to the district, provincial, and national levels. Feedback to the periphery occurs typically through the case review monthly meetings with districts teams, as mentioned above, and includes other stakeholders. However, when urgent this feedback will occur more rapidly.

#### Conflict zone

Since 1995, average ABER was higher in conflict districts. Conflict districts had an average ABER of 9.9% in 1995, compared to 5.4% in non-conflict districts. Conflict districts nearly doubled their ABER to 18.5% by 2000 while non-conflict districts only had a minor increase to 7.9%. By 2009, ABER decreased to 10.4% in conflict districts and 4.2% in non-conflict districts in 2009.

Conflict districts in some years had a slightly higher average per capita rate of ACD testing because the mobile clinics targeted hard to reach, at-risk populations, a majority of them located in the conflict areas. The per capita rate of ACD testing in conflict districts peaked in 2008 at 6.3% when 107,629 blood films were taken by these clinics. That year, 0.4% of the population in non-conflict districts was tested through ACD clinics.

In conflict districts, RMOs and their staff remained in their posts and were provided with vehicles and RDTs to conduct mobile clinics whenever it was safe to do so. There were reports of LTTE members assisting with and as beneficiaries of ACD clinics. In addition, the Sri Lankan Red Cross, the International Committee of the Red Cross (ICRC), and Medecins Sans Frontieres (MSF) assisted in providing diagnosis and treatment services. In addition, a Sri Lankan private not-for-profit organization, Tropical and Environmental Diseases and Health Associates (TEDHA), trains and deploys microscopists to APCD facilities in previous conflict districts as part of the Global Fund Round 8 grant, contributing to the scale-up of surveillance since 2009.

### Entomological surveillance

An entomological surveillance system was created in Sri Lanka shortly after the 1934–1935 epidemic, aiming to forecast increases in seasonal transmission and potential epidemics through identification of changes in vector breeding [3]. Trained mosquito collectors collected larvae and adult mosquitoes in dwellings on a monthly schedule. In 1940 the programme added mandatory inspections of rivers and streams for larvae by public health inspectors in each jurisdiction [3].

These activities continue today at both the central and district level. Routine pyrethrum spray collections in dwellings, cattle-baited net and hut trap collections, window trap collections, and larval mosquito surveys are conducted in malarious districts at pre-determined sentinel sites. Susceptibility tests and bio assays detect evidence of insecticide resistance. Data obtained from these tests are used in planning IRS and in ITN/LLIN distribution. Since 2008, TEDHA has also conducted entomological surveillance in its target districts.

Entomological surveillance in Sri Lanka serves two major purposes – it is part of the epidemic forecasting system and is also an essential component of the national integrated vector management (IVM) strategy [49]. IVM is used as a management tool in Sri Lanka and has been successful in agricultural areas through a combination of IRS, ITNs/LLINs, and larviciding and has contributed to the reduction in incidence. IVM in Sri Lanka brings together relevant sectors, community engagement and vector surveillance research to inform the use of insecticides and to determine the most appropriate mix of vector control interventions, environmental management and larval control. IVM in Sri Lanka began in the 1970s, when the hydroelectric and irrigation development project of Mahaweli River led to increases in malaria transmission [50]. Vector control and larval source management were used in response, with participation of communities and with involvement of the irrigation and agriculture sectors [50], [51]. In the late 2000s, Farmer Field Schools were used as a platform to make the connection between vector control for health and agriculture, educating farmers about the relationship between public health and agriculture, and involving them in vector management activities [52].

### Indoor residual spraying

Indoor residual spraying (IRS) was introduced in 1945 and became the primary vector control tool in Sri Lanka, where perennial spraying targeted all households in malarious districts. Following WHO recommendations issued in the mid-1990s [53], the AMC Directorate initiated a targeted spraying programme, focusing on historical areas of transmission, higher proportion of *P. falciparum*, chloroquine-resistant confirmed infections, and proximity to vector breeding sites [54]. A spatial mosaic insecticide rotation was then implemented in 1998, using a combination of up to six insecticides of two classes, organophosphates and pyrethroids. For example, in 2004, one zone of Kurunegala District applied Fenitrothion (organophosphate), while a neighboring sub-district used Cyfluthrin (pyrethroid). This spatial insecticide rotation has continued to today, unless there are delays in delivery of IRS supplies. The AMC instituted case-based and focal outbreak spraying in 2008, as a result of declining incidence.

In 1975 DDT was replaced by Malathion as reports of resistance to DDT increased. The first synthetic pyrethroid, Lambda-cyhalothrin, was introduced in 1994 and other new insecticides followed. The pyrethroid introduction may have increased community acceptance, which was already considered high (90% found in one study area for Malathion), as they emit less odor and do not leave visible residue on house walls [55], [56]. In 2002, Malathion was taken out of use because of mounting evidence of resistance.

National IRS coverage (estimated coverage of the population at risk) fluctuated over the 15-year period, from 64.8% (1995) to 46.5% (2000), then back down to 22.5% in 2005. In 2008, with the declining API there was a shift to case-based and focal outbreak IRS. By 2010, national coverage was down to 5.9% of the population at risk.

#### Conflict zone

The AMC Directorate conducted IRS in conflict and LTTE-controlled districts, notwithstanding the challenges, including risk of landmines. LTTE personnel assured the AMC Directorate that support would be given to malaria control measures in their zones – partly because their combatants were severely affected by malaria. RMOs in neighboring stable districts report that they assisted conflict districts throughout all years by coordinating IRS along and at times over the border. The government sent supplies, including insecticides, to conflict districts by requesting permission from the Ministry of Defense to send shipments via the sole accessible road to the northeast or, alternatively, by passenger ship. LTTE and AMC Directorate communication increased during the ceasefire period, from 2002 to 2006. It is likely that supply delivery became easier during this period of relative calm.

This communication and collaboration allowed for the continuation of IRS in the conflict zone. In 1995, the population at risk protected by IRS reached 23.5% in the conflict districts, while higher coverage of 79.6% was estimated for non-conflict districts. However, the population at risk protected in conflict districts increased to 52.2% (2000) and 45.9% (2005). This was a higher level of coverage than in non-conflict districts in 2000 (43.7%) and 2005 (10.9%).

### Insecticide-treated nets

The second primary vector control tool in Sri Lanka after IRS is the distribution of ITNs and LLINs. ITNs were distributed since 1999 and LLINs were introduced in 2004 with support from the Global Fund. Non-conflict districts were prioritized for ITN/LLIN distribution according to several factors: *P. falciparum* percentage in the past three years, mortality, number of pregnant women and children affected by malaria, proximity to a mosquito breeding site, and presence of internally displaced persons (IDPs) or migrant populations. Because security conditions changed frequently in conflict zones with associated displacement of populations, there was no formal stratification process for ITN/LLIN distribution.

In 2005, 14.8% of the population at risk was estimated to be covered by a LLIN, rising to 22.7% by 2009 and to 34.6% in 2010. Coverage estimates are based on an average three-year lifespan of an effective LLIN and assumes appropriate use. A study conducted in 2008 on use of LLINs found that a range of 89.6% to 90.9% of respondents slept under a LLIN [57].

#### Conflict zone

ITNs/LLINs were a key tool in conflict districts because of their higher caseloads, IDPs, and logistical challenges in conducting IRS. The Global Fund Round 1 grant supported the distribution of LLINs in conflict districts. The Ministry of Health, as part of the Global Fund grants, collaborated with a Sri Lankan NGO, Lanka Jatika Sarvodaya Shramadana Sangamaya (Sarvodaya), in the distribution of LLINs in northern conflict districts. The United Nations Children's Fund (UNICEF) and WHO also distributed LLINs. Through this network, 38.1% of the population at risk in conflict districts was covered by an LLIN in 2005, and 3.3% were covered in non-conflict districts. By 2009 coverage was similar in conflict districts (40.9%) and had increased in non-conflict districts (19.1%).

### Treatment and prophylaxis

Since the mid-1990s, it was recommended that all fever patients were to be tested for malaria. Since 2007, testing is recommended only for fever cases with malaria-related history and symptoms - body aches, joint pain, headache, nausea, vomiting, or diarrhea.

Sri Lanka has a national health service, and consultation and treatment are provided free at all public hospitals. Global Fund support allowed for the scale-up of diagnosis and treatment of malaria. In addition, travelers to endemic countries receive free chemoprophylaxis for up to three months, based on destination of travel.

From the mid-1990s until 2006, chloroquine and primaquine (0.25 mg/kg/day for adults), with a five-day regimen in endemic areas and 14 days in low transmission areas, was used for *P. vivax.* To ensure radical cure, or parasite clearance from both the blood and liver stages, a mandatory 14-day primaquine course was extended nationwide in 2006 [58]. Prevalence of Glucose-6-phosphate-dehydrogenase (G6PD) deficiency is relatively low (range of 1%–3%) [59]. Patients are not routinely screened for G6PD deficiency before treatment.

In 1999, it was estimated that 51% of *P. falciparum* infections were resistant to chloroquine, and by 2004 several cases of resistance to sulphadoxine-pyremethamine were detected [58]. As part of the malaria elimination strategy and as a result of an increased number of imported *P. falciparum* infections, artemisinin-based combination therapy (ACT), artemether-lumefantrin, was introduced in 2008. Primaquine for treatment of the gametocyte stage of the parasite has been documented to have been used in Sri Lanka since 1956 or earlier [3]. The National Treatment Guidelines recommend that all *P. falciparum* patients are admitted for three days, and *P. vivax* patients should receive follow-up visits to ensure compliance with the primaquine regimen [60].

While reports in the early 1990s indicated that self-treatment was common [59], more recent studies describe a low level of self-treatment for malaria and patient preference for confirmed diagnosis [61]–[63].

### Cost of malaria control and elimination

The Sri Lankan government and the Global Fund were and currently are the main sources of funding for malaria control in Sri Lanka. Funding for malaria control at the district level, based on risk and available resources, is allocated by the Ministries of Finance and Health. Sri Lanka successfully applied for and received funding for its malaria programme from the Global Fund in Rounds 1, 4, and 8. The approved grant amount was US $7.3 million in Round 1, $3.7 million in Round 4, and $21.6 million in Phase 1 of Round 8. The AMC Directorate, in collaboration with the Ministry of Health and the Global Fund Country Coordinating Mechanism Sri Lanka, determines which districts to include in grant proposals. Of the two districts selected for detailed costing studies, Anuradhapura received Global Fund support in Rounds 1 and 8, and Kurunegala in Rounds 4 and 8. WHO and World Bank/IDA provided additional support, which is only partially represented in the expenditure data. Both of these districts are located outside of the previous conflict zone, where major investments are targeted to scale up surveillance under the Global Fund Round 8 grant.

**Figure 6:**
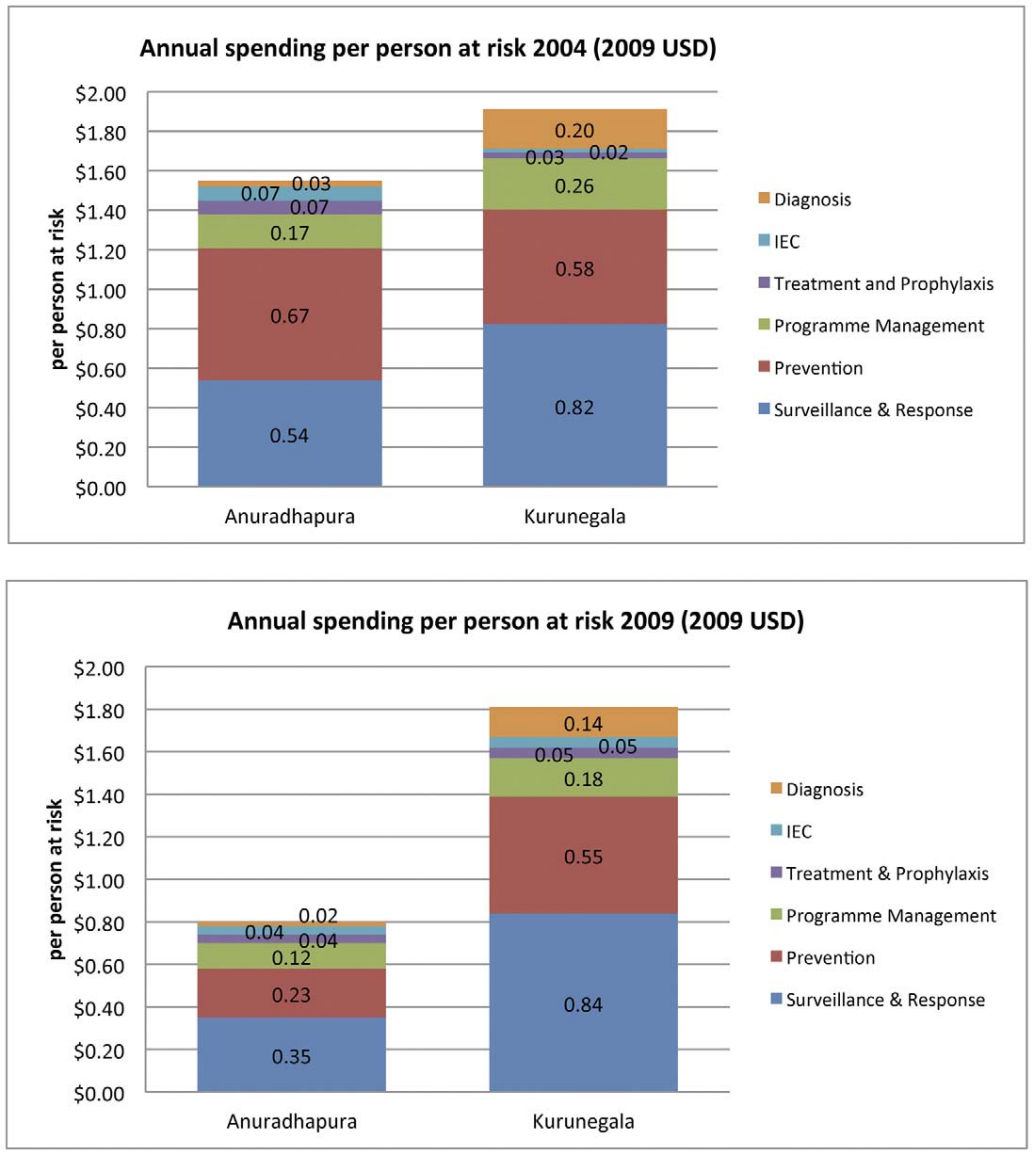
Costs per person at risk in 2004 and 2009 by intervention category, in $USD, two districts.

From 2004 to 2009, Anuradhapura District reported a decline of 48% in malaria programme expenditure per person at risk (Figure 6). Expenditures in Kurunegala District did not significantly change.

From 2004 to 2009, there were also differences between Anuradhapura and Kurunegala in the proportion of total expenditure allocated to programme components. The proportion allocated for prevention declined in Anuradhapura from 2004 to 2009 (43.6% to 29.1%), while the percentage for surveillance slightly increased. In contrast, the proportion of malaria expenditures in Kurunegala on prevention and surveillance measures stayed consistent over these years.

The proportion of total expenditure distributed across cost categories (e.g., consumables) shifted from 2004 to 2009 for both districts. Anuradhapura reported a slight decrease in proportion of expenditure on personnel from 2004 to 2009 (80.8% to 74.0%) while Kurunegala reported an increase in the proportion for personnel (48.3% to 67.5%). Nationally, malaria full-time employees decreased by 29% during this period, from 2,991 to 2,113 [64].

Initial cost estimates for elimination of *P. falciparum* and *P. vivax* in Sri Lanka, according to the five-year Global Fund Round 8 proposal budget projections, is $1 USD per person and $5 per person at risk [65].

## Discussion

### Principal findings

From 1999 to 2011, Sri Lanka achieved a 99.9% reduction in confirmed infections. API rapidly declined from 1999 (22.1) to less than 1 in 2004. Cases thereafter remained low, a trend found even during the post-tsunami period and more recently through PCR assay [39], [41], [42]. Deaths attributed to malaria also declined after 1998, with zero indigenous deaths since 2008.

As total malaria cases declined, the proportion caused by *P. vivax* increased. This trend has been identified in other countries with a declining malaria transmission [66], and may be linked to the successful treatment and vector control strategies that lower the *P. falciparum* burden faster than *P. vivax* [66], [67]. *P. vivax* is more challenging than *P. falciparum* to eliminate due to more asymptomatic and subclinical infections, infections at lower parasite densities making detection more difficult, the ability of the parasite cycle in the vector to exist at lower temperatures, and the existence of hypnozoites, the dormant liver stage that causes relapses. In addition, a 14-day treatment regimen with primaquine is required to kill hypnozoites. This radical cure of *P. vivax* complicates treatment adherence and has the side effect of hemolysis in some patients deficient with the G6PD enzyme [68]. A second trend seen in Sri Lanka is the substantial increase in proportion of malaria cases in adult males. This trend is related to a higher level of exposure in males of particular professions to infected vectors. In Sri Lanka, these at-risk populations tend to be gem miners and security personnel that work in remote jungle areas where access to medical treatment is difficult and use of preventive measures, such as domicile-based vector control methods, is more challenging.

The Anti-Malaria Campaign benefits from a long-running history rich in technical malaria control and elimination experience. The AMC, bolstered by external funding and partnerships with Sri Lankan and international NGOs, drove the decline through adaptation of innovative, evidence-based strategies of vector control, surveillance, and case management. IVM involved multiple sectors and communities in vector control, especially agricultural and irrigation sectors. IRS remained the primary vector control tool throughout the years, with new methods employed as they became available. The introduction of spatial mosaic insecticide rotation and new insecticide classes may have contributed to the effectiveness and acceptability of IRS. Coverage was maintained nationally.

The introduction of ITNs and, more recently, LLINs may have contributed to maintenance of low transmission by targeting areas with high transmission and hard-to-reach populations that may not have access to IRS. Collaborations with Sarvodaya, UNICEF, WHO and other partners made this distribution possible.

A strong passive case detection system, with a focus on malaria diagnosis and treatment through the APCD system, identifies the majority of malaria infections. Although coverage is relatively low, ACD is believed to help reduce the magnitude of peaks during transmission seasons by identifying both asymptomatic and symptomatic infections. Increased diagnostic capacity across the country over this period helped to sustain surveillance. The introduction of ACT and primaquine for *P. falciparum* and 14-day primaquine treatment for radical cure of *P. vivax* may have contributed to preventing onward transmission [67], [69].

Vector control and surveillance measures were maintained and at times scaled up more rapidly in districts having active conflict from 1995 to 2009. IRS was continued with support of government funding and LLINs were distributed to these areas through external funding and strong NGO partnerships. Starting in mid-2000, the annual rate of blood examination (ABER) was higher in the conflict areas. Targeted ACD increased access of remote populations to diagnosis and treatment and provided facilities to those whose health care infrastructure was damaged by conflict.

As the country moved from high endemicity in 2004 to controlled-low endemic transmission in 2009, Anuradhapura District reported a 48% reduction in expenditure. This decline may in part be due to decreases in external funding and to decreases in the scale of IRS activities. Increases in cost for elimination as compared to controlled low-endemic malaria were estimated for China, Mauritius, Swaziland, Tanzania-Zanzibar [70] and India [71], and Sri Lanka will likely have a similar experience. These costs will be extremely sensitive to the rate of importation and the degree to which costs can be shared with dengue and other vector-borne disease control efforts.

This case study adds to the growing body of literature that describes successful strategies to reduce malaria burden. Sri Lanka shares a number of success factors with other countries that have successfully reduced their burden, such as Bhutan, Brazil, Eritrea, India, and Vietnam, and with countries such as Mauritius who have successfully eliminated [72]–[74]. Bhutan, a fellow eliminating country, has seen a similar decline in cases, as well as an increase in the proportion of infections in adult males and in those caused by *P. vivax*. Bhutan and Sri Lanka both increased access to health services in a period of economic development, both of which likely contributed to success in driving down malaria. Both countries sustained malaria interventions, such as improved case management and vector control through IRS and LLIN. Similar to Brazil, Eritrea, India and Vietnam, Sri Lanka has a decentralized health system, yet the AMC Directorate maintains strong technical leadership of the programme. Sri Lanka also has had pockets of high transmission and maintained a similar approach, focusing on case management while introducing evidence-based prevention measures and further targeting of IRS. In financing, the country has benefited from World Bank funding and, more recently, Global Fund inputs which have assisted the country in providing the best intervention mix. The history of malaria elimination in Mauritius echoes the resurgence that occurred in Sri Lanka in 1963. Mauritius successfully eliminated malaria in 1998 for a second time, and has put significant resources toward maintaining malaria-free status. The Mauritius experience provides lessons for Sri Lanka and other eliminating countries.

In contrast to these countries, however, Sri Lanka achieved success despite having had a major long-running civil conflict. This success has been seen in a handful of other countries, such as in Afghanistan, Iraq, and Timor Leste [75], [76]. Timor Leste, similar to Sri Lanka, sustained malaria control in populations affected by war. Both countries adapted to the changing context and conducted case management and vector control measures at a scale large enough to avoid major outbreaks. The Sri Lanka case study shows that progress towards elimination can be achieved in conflict settings by maintaining malaria prevention and surveillance measures in conflict zones.

### Limitations

This case study relies upon national surveillance data to identify trends in malaria epidemiology. This case study did not include measures that estimate the number of infections that were unreported or in those that did not seek treatment.

Data from local and international NGOs or private clinics that participated in malaria control, prevention, diagnosis and treatment were not represented in this case study. Likewise, the costing analysis did not account for expenditures through non-governmental channels, or private expenditures by households. Costing data was collected and analyzed from a small sample of two districts and while the costs cannot reflect those of the entire country, they provide a basis for comparison across phases in the same district and may serve as a comparison against each other. An estimate for the cost of elimination was found in a previous analysis, which was based on budget projections, not expenditure data.

The interviews were conducted through a purposeful sampling methodology, with initial contacts supplied by the programme manager. There may be selection bias in the results of these interviews as a result of this selection process. However, the case study includes a wide range of positions and experiences in the interview participants, from programme managers to technical officers to IRS spraymen. This range is important to capture in order to reflect experience from decision-makers to those closest to the work.

### The way forward

Sri Lanka is working to eliminate malaria by the end of 2014 using surveillance, reporting, radical cure, rapid case response and case follow-up, and the management of imported malaria. In order to develop and implement effective strategies for elimination and prevention of reintroduction, countries such as Sri Lanka would benefit from further documentation of successful strategies, in particular around maintaining robust and efficient surveillance and response systems and engaging other sectors. Most importantly, Sri Lanka must continue to identify and treat imported malaria infections [77]. The risk of importation is likely to increase each year. Tourism revenues increased from 2009 to 2010 by 38% [25], [78]. Even more importantly, large ferry services have restarted from Tamil Nadu, India, to Colombo and smaller boat traffic between the countries is likely to increase in the coming years [79].

Also of importance is the assurance of long-term, sustainable funding. The recent cancellation of Round 11 from the Global Fund shows that support for malaria programmes, in particular low-burden countries, is at risk [80]. Reductions in funding contributed to the devastating resurgence in Sri Lanka in the 1960s and a repetition of this history must be avoided. A case must be made for continuing investment in Sri Lanka and in other low-endemic and elimination settings. Countries can better state this case if armed with high-quality cost estimates of elimination and prevention of reintroduction. Comprehensive cost-benefit analyses using a macro-economic framework [81], taking into account well-described and quantified benefits [8], will enhance this argument.

## Supporting Information

Appendix S1 **A Desk Review of Literature on Malaria Control and Elimination in Sri Lanka.**
(DOCX)
