# Supplementary material for: Malaria Control and Elimination in Sri Lanka: Documenting Progress and Success Factors in a Conflict Setting
Source: PLoS One. 2012 Aug 29;7(8):e43162. doi: 10.1371/journal.pone.0043162 (PMC3430652; doi:10.1371/journal.pone.0043162)
Supplement: Appendix S1 — A Desk Review of Literature on Malaria Control and Elimination in Sri Lanka. (DOCX) [file pone.0043162.s001.docx]

**Appendix S1: A Desk Review of Literature on Malaria Control and Elimination in Sri Lanka**

The desk review identified the following publications and grey documents relating to Sri Lanka and malaria control and elimination.

**Publications on malaria control and elimination in Sri Lanka (112)**

Abeyaratne KP, Premawansa S, Rajapakse L, Roberts DF, Papiha SS (1976) A survey of glucose-6-

phosphate-dehydrogenase deficiency in the North Central Province of Sri Lanka (formerly Ceylon). American Journal of Physical Anthropology 44: 135-138.

Abeysekera T, Wickremasinghe AR, Gunawardena DM, Mendis KN (1997) Optimizing the malaria data

recording system through a study of case detection and treatment in Sri Lanka. Tropical Medicine and International Health 2: 1057-1067.

Akhtar R, Dutt AK, Wadhwa V (Eds.) (2010) Resurgence of Malaria in Sri Lanka in the 1970s. Advances

in Asian Human-Environmental Research: Malaria in South Asia 1: 29-41.

Amarasiri de Silva MW, Wijekoon A, Hornik R, Martines J (2001) Care seeking in Sri Lanka: one possible

explanation for low childhood mortality. Social Science & Medicine 53: 1363-1372.

Amerasinghe FP, Amerasinghe PH, Malik Peiris JS, and Wirtz RA (1991) Anopheline ecology and malaria

infection during the irrigation development of an area of the Mahaweli Project, Sri Lanka. Am J Trop Med Hyg 45: 226-235.

Amerasinghe PH, Amerasinghe FP (1999) Multiple host feeding in field populations of Anopheles

culicifacies and An. subpictus in Sri Lanka. Medical & Veterinary Entomology 13: 124-131.

Amerasinghe PH, Amerasinghe FP, Konradsen F, Fonseka KT, Wirtz RA (1999) Malaria vectors in a

traditional dry zone village in Sri Lanka. Am J Trop Med Hyg 60: 421-429.

Amerasinghe PH, Amerasinghe FP, Wirtz RA, Indrajith NG, Somapala W et al. (1992) Malaria

Transmission by Anopheles subpictus (Diptera: Culicidae) in a New Irrigation Project in Sri Lanka. Journal of Medical Entomology 29: 577-581.

Asoka C, Gamage-Mendis JR, Carter R, Mendis KM (1991) Infectious reservoir of

Plasmodium vivax and Plasmodium falciparum malaria in an endemic region of Sri Lanka. Am J Trop Med Hyg 45: 479-487.

Attanayake N (1994) Cost effectiveness of anti-malaria activities in Sri Lanka. London School of Hygiene

and Tropical Medicine.

Baird JK (2007) Neglect of Plasmodium vivax malaria. Trends in Parasitology 23: 533-539.

Barik TK, Sahu B, Swain V (2009) A review on Anopheles culicifacies: From bionomics to control with

special reference to Indian subcontinent. Acta Tropica 109: 87-97.

Beier J, Keating J, Githure J, Macdonald M, Impoinvil D et al. (2008) Integrated vector management for

malaria control. Malaria Journal 7: S4.

Bouffard S, Carment D (2006) The Sri Lanka Peace Process. Journal of South Asian Development 1: 151-

177.

Briët OJT, Vounatsou P, Amerasinghe, PH (2008) Malaria seasonality and rainfall seasonality in Sri

Lanka are correlated in space. Geospatial Health 2: 183-190.

Briët OJT, Galappaththy G, Amerasinghe P, Konradsen F (2006) Malaria in Sri Lanka: one year post-

tsunami. Malaria Journal 5: 42.

Briët OJT, Vounatsou P, Gunawardena D, Galappaththy G, Amerasinghe P (2008) Models for short term

malaria prediction in Sri Lanka. Malaria Journal 7: 76.

Briët OJT, Vounatsou P, Gunawardena D, Galappaththy G, Amerasinghe P (2008) Temporal correlation

between malaria and rainfall in Sri Lanka. Malaria Journal 7: 77.

Briet OJT, Gunawardena DM, van der Hoek W. Amerasinghe FP (2003) Sri Lanka Malaria Maps. Malaria

Journal 2: 1-10.

Briet OJT, Galappaththy GNL, Konradsen F, Amerasinghe PH, Amerasinghe FP (2005) Maps of Sri Lanka

malaria situation preceding the tsunami and key aspects to be considered in the emergency phase and beyond. Malaria Journal 4: 1-11.

Bruce-Chwatt L, Garrett-Jones C, Weitz B (1966) Ten years' study (1955-64) of host selection by

anopheline mosquitos. Bulletin of the World Health Organization 35: 405.

Casman E, Dowlatabadi H, Eds (2002) The Contextual Determinants of Malaria. pp 1-381.

Cohen JM, Smith DL, Vallely A, Taleo G, Malefoasi G et al. (2009) Holding the Line. In: Feachem,

RGA, Phillips AA, Targett GA (eds) Shrinking the Malaria Map: A Prospectus on Malaria

Elimination San Francisco: The Global Health Group, Global Health Sciences, University of California, San Francisco.

Cohen J, Moonen B, Snow R, Smith D (2010) How absolute is zero? An evaluation of historical and

current definitions of malaria elimination. Malaria Journal 9: 213.

Cohen JM, Smith DL, Cotter C, Ward A, Yamey G et al. (2011) Malaria resurgence: a systematic review

and assessment of its causes. Malaria Journal 11: 122.

Cohn S (1973) Assessing the costs and benefits of anti-malaria programs: The Indian Experience.

American Journal of Public Health 63: 1086-1096.

Dunn CL (1936) Malaria in Ceylon. An enquiry into its causes. Postgrad Med J 12: 483.

Klinkenberg E, van der Hoek W, Amerasinghe FP (2004) A malaria risk analysis in an irrigated area in Sri

Lanka. *Acta Tropica* 89: 215-225.

Feachem RGA, Phillips AA, Targett GA (eds) (2009) Shrinking the Malaria Map: A Prospectus on Malaria

Elimination pp 1-187.

Feachem RGA, Phillips AA, Targett GA, Snow RW (2010) Call to action: priorities for malaria elimination.

*The Lancet* 376: 1517-1521.

Feachem RGA, Phillips AA, Hwang J, Cotter C, Wielgosz B et al. (2010) Shrinking the Malaria Map:

Progress and Prospects. *The Lancet* 367: 1566-1578.

Fernando S, Abeyasinghe R, Galappaththy G, Gunawardena N, Rajapakse L (2008) Community factors

affecting long-lasting impregnated mosquito net use for malaria control in Sri Lanka. Transactions of the Royal Society of Tropical Medicine and Hygiene 102: 1081-1088.

Fernando SD, Abeyasinghe RR, Galappaththy GNL, Rajapaksa LC (2009) Absence of Asymptomatic

Malaria Infections in Previously High Endemic Areas of Sri Lanka. Am J Trop Med Hyg 81: 763-767.

Fernando SD, Karunaweera ND, Fernando WP (2004) Evaluation of a rapid whole blood

immunochromatographic assay for the diagnosis of Plasmodium falciparum and Plasmodium vivax malaria. The Ceylon Medical Journal 49: 7-11.

Fernando W (2002) Hydraulic civilization and malaria: challenges faced by the malaria control program

of Sri Lanka. Malaria in Irrigated Agriculture: 29.

Fernando WP, Ratnapala PR (1988) "Report on Research Project Funded by the International Science

and Technology Institute, 'A survey to ascertain the prevalence of G-6-PD enzyme deficiency in Sri Lanka, and its geographical and ethnical distribution.' " Colombo: International Science and Technological Institute.

Fonseka J, Mendis KN (1987) A metropolitan hospital in a non-endemic area provides a sampling pool

for epidemiological studies on vivax malaria in Sri Lanka. Transactions of the Royal Society of Tropical Medicine and Hygiene 81: 360-364.

Frederiksen H (1960) Malaria Control and Population Pressure in Ceylon. Public Health Reports 75: 865-

868.

Frederiksen H (1961) Determinants and Consequences of Mortality Trends in Ceylon. Public Health

Reports 76: 659-664.

Galappaththy GNL (1999) Elimination of P. falciparum malaria from Sri Lanka: an epidemiological

possibility. Journal of the College of Community Physicians Sri Lanka, 4.

Gill CA (1936) The mode of onset of the malaria epidemic in Ceylon. Transactions of the Royal Society of

Tropical Medicine and Hygiene 30: 101-107.

Gill CA (1936) Some points in the malaria of Ceylon. Annals of Tropical Medicine and Parasitology 91:

428-480.

The Global Health Group and the Malaria Atlas Project (2011) The Atlas of the Asia Pacific Malaria

Elimination Network, 2011 San Francisco: The Global Health Group, Global Health Sciences, University of California, San Francisco.

Gray RH (1974) The Decline of Mortality in Ceylon and the Demographic Effects of Malaria Control.

Population Studies 28: 205-229.

Green CA, Miles SJ (1980) Chromosomal evidence for sibling species of the malaria vector Anopheles

culicifacies Giles. Journal of Tropical Medicine and Hygiene 83: 75-78.

Gunawardena DM, Wickremasinghe AR, Muthuwatta L, Weerasingha S, Rajakaruna J et al. (1998)

Malaria risk factors in an endemic region of Sri Lanka, and the impact and cost implications of risk factor-based interventions. Am J Trop Med Hyg 58: 533-542.

Hapuarachchi HAC, Dayanath MYD, Abeysundara S, Bandara KBAT, Abeyewickreme W et al. (2004)

Chloroquine resistant falciparum malaria among security forces personnel in the Northern Province of Sri Lanka. Ceylon Medical Journal 49: 47-51.

Hsiang M, Abeyasinghe RR, Whittaker M, Feachem RGA (2010) Malaria elimination in Asia-Pacific:

An under-told story. The Lancet 375: 1586-1587.

International Crisis Group (2006) Sri Lanka: The Failure of the Peace Process. Asia Report Number 124.

Available: <http://www.crisisgroup.org/~/media/Files/asia/south-asia/sri-lanka/124_sri_lanka___the_failure_of_the_peace_process.pdf>. Accessed November 15, 2011.

Jayawardena HKWI, Sonnadara DUJ, Jayewardene DR (2005) Trends of Rainfall in Sri Lanka over the Last

Century. Sri Lankan Journal of Physics 6: 7-17.

Jones M (2000) The Ceylon Malaria Epidemic of 1934-1935: A Case Study in Colonial Medicine. The

Society for the Social History of Medicine 13: 24.

Kahn J, Basu S, Boyle C, Hsiang MS, Jamison DT et al. (2009) Financing elimination In: Feachem RGA,

Phillips AA, Targett GA Shrinking the malaria map: a prospectus on malaria elimination San Francisco: University of California, San Francisco; 2009: 61-80.

Kannathasan S, Antonyrajan A, Srikrishnaraj KA, Karunaratne SHPP, Karunaweera ND et al. (2008)

Studies on prevalence of anopheline species and community perception of malaria in Jaffna district, Sri Lanka. J Vector Borne Dis 45: 231-239.

Karunaratne SHPP, Hemingway J (2001) Malathion resistance and prevalence of malathion

carboxylesterase mechanism in populations of mosquito vectors of disease in Sri Lanka. Bulletin of the World Health Organization 79: 1060-1064.

Karunaratne WA (1959) The Influence of Malaria Control on Vital Statistics in Ceylon. The Journal of

Tropical Medicine and Hygiene 62: 79-85.

Kelly-Hope L, Yapabandara A, Wickramasinghe M, Perera M, Karunaratne S et al. (2005) Spatiotemporal

distribution of insecticide resistance in Anopheles culicifacies and Anopheles subpictus in Sri Lanka. Transactions of the Royal Society of Tropical Medicine and Hygiene 99: 751-761.

Konradsen F, Steele P, Perera D, van der Hoek W, Amerasinghe PH et al. (1999) Cost of Malaria Control

in Sri Lanka. Bulletin of the World Health Organization 77: 301-309.

Konradsen F, van der Hoek W, Amerasinghe PH, Amerasinghe FP, Fonseka KT (1997) Household

responses to malaria and their costs: a study from rural Sri Lanka. Transactions of the Royal Society of Tropical Medicine and Hygiene 91: 127-130.

Kusumawathie PHD, Wickremasinghe AR, Karunaweera ND, Wijeyaratne MJS (2008) Larvivorous

Potential of the Guppy, Poecilia reticulata, the Anopheline Mosquito Control in Riverbed Pools Below the Kotmale Dam, Sri Lanka. Asia-Pacific Journal of Public Health 20: 56-63.

Kusumawathie PHD, Wickremasinghe AR, Karunaweera ND, Wijeyaratne MJS (2008) Costs and

effectiveness of application of Poecilia reticulata (guppy) and temephos in anopheline mosquito control in river basins below the major dams of Sri Lanka. Transactions of the Royal Society of Tropical Medicine and Hygiene 102: 705-711.

Langford CM (1996) Reasons for the decline in mortality in Sri Lanka immediately after the Second

World War: a re-examination of the evidence. Health Transition Review 6: 3-23.

Martins JS, Zwi AB, Martins N, Kelly PM (2009) Malaria control in Timor-Leste during a period

of political instability: what lessons can be learned? Conflict and Health 3:11.

Mattys B, Sherkanov T, Karimov SS (2008) History of malaria control in Tajikistan and rapid malaria

appraisal in an agro-ecological setting. Malaria Journal 7: 217.

McCombie SC (1996) Treatment seeking for malaria: A review of recent research. Soc Sci Med 43: 933-

945.

Meegama SA (1967) Malaria Eradication and its Effect on Mortality Levels. Population Studies 21: 207-

237.

Mendis C, Gamage-Mendis AC, De Zoysa APK, Abhayawardena TA, Carter R et al. (1990) Characteristics

of malaria transmission in Kataragama, Sri Lanka: A focus for immuno-epidemiological studies. Am J Trop Med Hyg 42: 298-308.

Mendis K, Munesinghe YD, de Silva YNY, Keragalla I, Carter R (1987) Malaria Transmission-

Blocking Immunity Induced by Natural Infections of Plasmodium vivax in Humans. Infection and Immunity 55: 369-372.

Mendis K, Sina BJ, Marchesini P, Carter R (2001) The neglected burden of Plasmodium vivax malaria. Am

J Trop Med Hyg 64: 97-106.

Mills A, Lubell Y, Hanson K (2008) Malaria eradication: the economic, financial and institutional

challenge. Malaria Journal 7: S11.

Morgan T (1956) The Economic Development of Ceylon. The Annals of the American Academy: 92-100.

184.

Pavilupillai JJ, Sangaralingam D, Muthuladchumy V, Sinnathamby NS, Ramasamy R (2010)

Anopheles culicifacies breeding in brackish waters in Sri Lanka and implications for malaria

control. Malaria Journal 9: 106.

Perera M, Hemingway J, Karunaratne SP (2008) Multiple insecticide resistance mechanisms involving

metabolic changes and insensitive target sites selected in anopheline vectors of malaria in Sri Lanka. Malaria Journal 7: 168.

Pinikahana J, Dixon RA (1993) Trends in Malaria Morbidity and Mortality in Sri Lanka. Indian journal of

malariology 30: 51-55.

Price RN, Tjitra E, Guerra CA, Yeung S, White NJ et al. (2007) Vivax Malaria: Neglected and Not Benign.

Am J Trop Med Hyg 77: 79-87.

Ramasamy R, de Alwis R, Wuesundere A, Ramasamy MS (1992) Malaria transmission at a new irrigation

project in Sri Lanka: The emergence of Anopheles annularis as a major vector. Am J Trop Med Hyg 47: 547-553.

Rajakaruna R, Amerasinghe P, Galappaththy GNL, Konradsen F, Briet O (2009) Current Status of Malaria

and Anti-Malarial Drug Resistance in Sri Lanka. Ceylon Journal of Science (Biological Sciences) 37: 15.

Rajakaruna R, Weerasinghe M, Alifrangis M, Amerasinghe P, Konradsen F (2006) The role of private drug

vendors as malaria treatment providers in selected malaria endemic areas of Sri Lanka. Journal

of Vector Borne Diseases 43: 58.

Rajakaruna RS, Alifrangis M, Amerasinghe PH, Konradsen F (2010) Pre-elimination stage of malaria

in Sri Lanka: assessing the level of hidden parasites in the population. Malaria Journal 9: 25.

Rajendram S, Cadek MHMA, Visvalingam T (1950) Malaria Eradication in Ceylon. Nature 166: 486-486.

Rajendram S, Jayewickreme SH (1951) Malaria in Ceylon. Part I. The control and prevention of epidemic

malaria by the residual spraying of houses with D.D.T. Indian journal of malariology 5: 1-73.

Rajendram S, Jayewickreme SH (1951) Malaria in Ceylon. Part II. The control of endemic malaria at

Anuradhapura by the residual spraying of houses with D.D.T. Indian journal of malariology 5: 75-124.

Ramasamy MS, Nagendran K (1995) The intermediate rainfall zone as a source of malaria epidemics in

Sri Lanka. Ceylon Medical Journal 40: 44-52.

Ramasamy MS, Kulasekera R, Srikrishnaraj KA, Ramasamy R (1994) Population dynamics of

anthropophilic mosquitoes during the northeast monsoon season in the malaria epidemic zone of Sri Lanka. Medical and Veterinary Entomology 8: 265-274.

Ramos WM, Sardinha JFJ, Costa MRF, Santana MS, Alecrim MGC et al. (2010) Clinical aspects of

hemolysis in patients with P. vivax malaria treated with primaquine, in the Brazilian Amazon. Brazilian Journal of Infectious Diseases 14: 410-412.

Reilley B, Abeyasinghe R, Pakianathar MV (2002) Barriers to prompt and effective treatment of malaria

in northern Sri Lanka. Tropical Medicine and International Health 7: 744-749.

Roberts DF, Papiha SS, Abeyaratne KP (1972) Red Cell Enzyme Polymorphisms in Ceylon Sinhalese. Amer

J Hum Genet 24: 181-188.

Roberts DR, Manguin S, Mouchet J (2000) DDT house spraying and re-emerging malaria. Lancet 356:

330-332.

Rojas W, Penaranda F, Echavarria M (1992) Strategies for Malaria Control in Colombia. Parasitology

Today 8: 141-144.

Russell S (2005) Treatment-seeking behaviour in urban Sri Lanka: Trusting the state, trusting private

providers. Social Science & Medicine 61: 1396-1407.

Sabot O, Cohen JM, Hsiang MS, Kahn JG, Basu S et al. (2010) Costs and Financial Feasibility of Malaria

Elimination. The Lancet 376: 1604-1615.

Samarasinghe L (1992) Malaria control program in Sri Lanka. Southeast Asian Journal of Tropical

Medicine and Public Health 23: 61-65.

Schousboe M, Rajakaruna R, Salanti A, Hapuarachchi H, Galappaththy GNL et al. (2007) Island-wide

diversity in single nucleotide polymorphisms of the Plasmodium vivax dihydrofolate reductase and dihydropteroate synthetase genes in Sri Lanka. Malaria Journal 6: 28.

Schulemann W (1936) The Malaria Epidemic in Ceylon. The British Medical Journal: 186.

Premawansa S, Gamage-Mendis A, Perera L, Begarnie S, Mendis K, Carter R (1994) Plasmodium

falciparum transmission-blocking immunity under conditions of low endemicity as in Sri Lanka. Parasite Immunology 16: 35-42.

Seo SN MR, Munasinghe M (2005) Climate Change and Agriculture in Sri Lanka: A Ricardian valuation.

Enviro and Dev Econ 10: 581-596.

Sivagnanasundram C (1973) Reproduction rates of infection during the 1967-1968 P. vivax malaria

epidemic in Sri Lanka (Ceylon). Unknown Unknown: 83-86.

Subbarao SK (1988) The Anopheles culicifacies complex and control of malaria. Parasitology Today 4: 72-

75.

Tatem JT, Smith DL, Gething PW, Kabaria CW, Snow RW, Hay SI (2010) Ranking of elimination feasibility

between malaria-endemic countries. The Lancet 376: 1579-1591.

Unknown (1967) Editorial: The "Resurrection" of Malaria. The Ceylon Medical Journal 12: 1-2.

van Bortel W, Harbach R, Trung H, Roelants P, Backeljau T et al. (2001) Confirmation of Anopheles

varuna in Vietnam, previously misidentified and mistargeted as the malaria vector Anopheles minimus. Am J Trop Med Hyg 65: 729-732.

van der Hoek W, Konradsen F, Perera D, Amerasinghe PH, Amerasinghe FP (1997) Correlation between

rainfall and malaria in the dry zone of Sri Lanka. Annals of Tropical Medicine and Parasitology 91: 945-949.

van der Hoek W, Konradsen F, Dijkstra S, Amerasinghe PH, Amerasinghe FP (1998) Risk factors for

malaria: a microepidemiological study in a village in Sri Lanka. Transactions of the Royal Society of Tropical Medicine and Hygiene 92: 265-269.

van der Hoek, Konradsen F, Amerasinghe PH, Perera D, Piyaratne MK, Amerasinghe FP (2003) Towards a

risk map of malaria for Sri Lanka: the importance of house location relative to vector breeding sites. International Journal of Epidemiology 32: 280-285.

von Seidlein L, Greenwood BM (2003) Mass administrations of antimalarial drugs. Trends in Parasitology

19: 452-460.

Wattavidanage J, Carter R, Perera KLRL, Munasingha A, Bandara S et al. (1999) TNFa*2 marks high risk of

severe disease during Plasmodium falciparum malaria and other infections in Sri Lankans. Clin Exp Immunol 115: 350-355.

Wells TNC, Burrows JN, Baird JK (2010) Targeting the hypnozoite reservoir of Plasmodium vivax: the

hidden obstacle to malaria elimination. Trends in Parasitology 26: 145-151.

Worth HN (1937) The control of Anopheline breeding in river beds. Transactions of the Royal Society of

Tropical Medicine and Hygiene, Issue 5, pp 521-530.

Wickremasinghe R, Galappaththy GNL, Fernando WAP, de Monbrison F, Wijesinghe RS et al. (2008) An

Indigenous Case of Plasmodium ovale Infection in Sri Lanka. Am J Trop Med Hyg 78: 206-207.

Wijesundera MS (1988) Malaria Outbreaks in New Foci in Sri Lanka. Parasitology Today 4: 147-150.

Yapabandara A, Curtis F (2002) Laboratory and field comparisons of pyriproxyfen, polystyrene beads

and other larvicidal methods against malaria vectors in Sri Lanka. Acta Tropica 81: 211-223.

Yapabandara AMGM, Curtis CF, Wickramasinghe MB, Fernando WP (2001) Control of malaria vectors

with the insect growth regulator pyriproxyfen in a gem-mining area in Sri Lanka. Acta Tropica 80: 265-276.

Zubair L, Galappaththy G, Yang H, Chandimala J, Yahiya Z et al. (2008) Epochal changes in the

association between malaria epidemics and El Niño in Sri Lanka. Malaria Journal 7: 140.

**WHO reports and documents (16)**

Berg H, van den KB (2006) Evaluation of the Integrated Pest Management and Vector Management

(IPVM) project in Sri Lanka. New Dehli: World Health Organization, South East Asia Regional Office: 1-46.

Carter R, Mendis K, Roberts D (2000) Spatial targeting of interventions against malaria. Bulletin of

the World Health Organization 78: 1401-1411.

Clarke JL, Herath PRJ, Wickramasinghe MB (1974) Studies on DDT Resistance in Anopheles culicifacies in

Sri Lanka. World Health Organization WHO/MAL/74.826.

Fernando WP, Silva KT, Telesinghe LD (1989) Relative importance of factors affecting the

receptivity of an agriculturally oriented community towards malaria control. WHO/TDR Final Report T16/181/SER/35A.

The Global Malaria Programme (2007) Malaria elimination: A field manual for low and moderate

endemic countries. 1-85. Geneva: The World Health Organization.

Gunaratna LF (1956) Recent Antimalaria Work in Ceylon. Bulletin of the World Health Organization 15:

791-799.

Najera J, Kouznetsov RL, Delacollette C (2010) Malaria Epidemics Detection and Control Forecasting and Prevention. Roll Back Malaria website. Available: <http://www.rollbackmalaria.org/docs/najera_epidemics/naj1.htm>. Accessed November 29, 2010.

WHO Malaria Conference for Western Pacific and South East Asia Regions (1954) Information on the

Malaria Control Programme in Ceylon. World Health Organization: WHO/Mal/103.14: 1-10.

WHO (2010) Guidelines for the treatment of malaria, Second Edition. Geneva: World Health

Organization.

WHO (2004) Global Strategic Framework for Integrated Vector Management. P. 1-12. Geneva: World

Health Organization.

WHO (1993) A global strategy for malaria control. Geneva: World Health Organization.

WHO (1995) Vector control for malaria and other mosquito-borne diseases, Report of a WHO Study

Group. WHO Technical Report Series 857. Geneva: World Health Organization.

WHO (2009) World Malaria Report 2009. Geneva: Global Malaria Programme.

WHO (2010) World Malaria Report, 2010. Geneva: Global Malaria Programme.

WHO (1961) Review of Quarterly Reports in Surveillance Operations in 1960. Geneva: World Health

Organization WHO/Mal/289.

van den Berg H, von Hildebrand A, Ragunathan V, Das PK (2007) Reducing vector-borne disease by

empowering farmers in integrated vector management. Bulletin of the World Health Organization 85: 561-566.

**Documents from Sri Lanka Ministry of Health, Anti-Malaria Campaign, or regarding external aid programmes implemented by the AMC or other organizations/agencies (56)**

ELECTRONIC FILES (19)

Abeyasinghe RR, Galappaththy GNL, Lengeler C (2010) Monitoring and evaluation Plan, 2010-2014 Draft.

Colombo: National Malaria Control Programme of Sri Lanka. Available: <http://www.malariacampaign.gov.lk/Downloads/MnE%20Book%2023032011.pdf>. Accessed June 10, 2012.

Anti-Malaria Campaign Directorate (2010). Estimated population at risk, all districts. Accessed July 30,

2010.

Anti-Malaria Campaign (1995-2008). Morbidity District-wise. Accessed December 15, 2009 from AMC

HQ Files, Colombo, Sri Lanka.

Anti-Malaria Campaign (1995 to 2008). Results of Blood Film Examination. Accessed December 15, 2009

from AMC HQ Files, Colombo, Sri Lanka.

Anti-Malaria Campaign (1997-2009). Microscopically-confirmed malaria cases in Sri Lanka. Accessed

December 15, 2009 from AMC Headquarter Files, Colombo, Sri Lanka.

Anti-Malaria Campaign (1997, 2006, 2007). Annual Report 2007. Accessed December 18, 2009 from

AMC Headquarter Files, Colombo, Sri Lanka.

Anti-Malaria Campaign (2001 - 2007). World Health Organization report: Information on Malaria Control

Programme for the Regional Programme. Accessed 2009 from AMC HQ Files, Colombo, Sri Lanka.

Anti-Malaria Campaign (2001, 2004 - 2009). Insecticide Usage/Spray Data. Accessed December 15, 2009

from AMC Headquarter Files, Colombo, Sri Lanka.

Anti-Malaria Campaign (2002). Administration Report of the Anti-Malaria Campaign, 2002. Accessed

December 18, 2009 from AMC Headquarter Files, Colombo, Sri Lanka.

Anti-Malaria Campaign (2002). Country Report on Progress of Malaria.

Anti-Malaria Campaign (2002-2008). Annual Health Bulletins. Accessed December 18, 2009 from AMC

Headquarter Files, Colombo, Sri Lanka.

Anti-Malaria Campaign (2003, 2004, 2006-07). Population, Mid-Year. Accessed December 15, 2009 from

AMC Headquarter Files, Colombo, Sri Lanka.

Anti-Malaria Campaign (2004-2006). Bed nets. Accessed December 15, 2009 from AMC Headquarter

Files, Colombo, Sri Lanka.

Anti-Malaria Campaign (2008). LLIN. Accessed December 15, 2009 from AMC Headquarter Files,

Colombo, Sri Lanka.

Anti-Malaria Campaign (1995 - 2008). Morbidity Sri Lanka, Monthly. Accessed December 15, 2009 from

AMC Headquarter Files, Colombo, Sri Lanka.

Anti-Malaria Campaign (1995 to 2008). Malaria Deaths. Accessed December 15, 2009 from AMC

Headquarter Files, Colombo, Sri Lanka.

Ministry of Defense, Sri Lanka (2010). The Spatial and Temporal Distribution of Terrorist Activities and

Government Humanitarian Efforts, 1981-2009. Available: http://wwwdefencelk/gis/Final_Final61swf: 1-4. Accessed September 8, 2010.

South Asia Terrorism Portal website. Available:

<http://www.satp.org/satporgtp/countries/shrilanka/database/index.html>). Accessed June 30, 2010.

South Asia Terrorism Portal (2010). Sri Lanka Datasheets: Fatalities District-Wise, 2005 to 2010.

Available: <http://wwwsatporg/satporgtp/countries/shrilanka/database/indexhtml>). Accessed

June 30, 2010.

HARD COPY FILES (37)

Abeyasinghe DRR (Unknown) Abstracts: Ecologically based Integrated Pest Management and Integrated

Vector Management: key elements of pesticide risk reduction strategies. Intergovernmental Forum on Chemical Safety, Global Partnerships for Chemical Safety, Contributing to the 2020 Goal: 1-13.

Anti-Malaria Campaign Directorate (2010) Summary Tablet Information. Colombo: Sri Lanka Ministry of

Health.

Anti-Malaria Campaign Directorate (2010) LLIN Data. Colombo: Sri Lanka Ministry of Health

Anti-Malaria Campaign Directorate (2010) Hudsons Spray Machines. Colombo: Sri Lanka Ministry of

Health

Anti-Malaria Campaign (2010) Parasitology Lab - Consumables and Capital Equipment. Colombo: Sri

Lanka Ministry of Health

Anti-Malaria Campaign (2010). Ampara - Pharma 1995 to 2009. Colombo: Sri Lanka Ministry of Health.

Anti-Malaria Campaign (2010). Anuradhapura - Pharma, 1995 to 2009. Colombo: Sri Lanka Ministry of

Health.

Anti-Malaria Campaign (2010). Kurunegala - Pharma, 1995 to 2009. Colombo: Sri Lanka Ministry of

Health.

Anti-Malaria Campaign (2005-2009). Physical Progress - Round 4 - Phase 2 - Year 4. Colombo: Sri Lanka

Ministry of Health

Anti-Malaria Campaign (2009). Programme Schedule of PHLT Training. Colombo: Sri Lanka Ministry of

Health

Anti-Malaria Campaign (2009). Programme Schedule of PHI Training. Colombo: Sri Lanka Ministry of

Health.

Anti-Malaria Campaign (2009) Programme Schedule of PHFO Training. Colombo: Sri Lanka

Ministry of Health.

Anti-Malaria Campaign (2008) Strategic Plan for Phased Elimination of Malaria 2008-2012.

World Bank (2002). Implementation Completion Report (IDA-29280) on a Credit in the Amount of SDR

13.0 Million (U.S. $18.8 Million equivalent) to the Democratic Socialist Republic of Sri Lanka for a Health Services Project. Washington DC: The World Bank.

Anti-Malaria Campaign (1995-2008) Annual Blood Examination Rate. Accessed 12/24/09 from AMC HQ

Files, Colombo, Sri Lanka.

Anti-Malaria Campaign (1999, 2003, 2007, 2009) Mefloquine Tabs (250mg). Accessed 12/24/09 from

AMC HQ Files, Colombo, Sri Lanka.

Anti-Malaria Campaign (2001, 2002, 2003, 2004). RDT Test Kits. Accessed 12/24/09 from AMC HQ Files,

Colombo, Sri Lanka.

Bandara S (2002) Trial of permethrin treated nets in malaria control in Southern Sri Lanka, PhD thesis,

Faculty of Medicine, University of Colombo.

Country Coordinating Mechanism, Sri Lanka. Round 4 Malaria grant proposal.

Country Coordinating Mechanism, Sri Lanka (2002) Grant Agreement, Malaria, Round 1.

Country Coordinating Mechanism, Sri Lanka (2008) Grant Proposal - Round 8 - Malaria.

Department of Census and Statistics, Sri Lanka (1996). Statistical Abstract of the Democratic Socialist

Republic of Sri Lanka. Colombo: Ministry of Finance and Planning.

Department of Census and Statistics, Sri Lanka (2000). Statistical Abstract 2000. Colombo: Ministry of

Finance and Planning.

Galappaththy GNL (2009). An overview: Drug resistance status of malaria in Sri Lanka.

Unpublished manuscript.

Global Fund (2004). Report of the Independent Evaluation of Phase-one (June 2003-June 2004) of the

Malaria Control Project in Sri Lanka, funded by the Global Fund for AIDS, TB, and Malaria (GFATM), SRI-102-G01-M-00 (Malaria Control).

Global Fund (2006). Report for the Independent Evaluation of the Global Fund for AIDS, TB, and Malaria

(GFATM) malaria control project (Round 4).

Global Fund (2007). Report of the Independent Evaluation of the Global Fund for AIDS, TB, and Malaria

(GFATM) malaria control project Round 1, Phase 2.

Global Fund (2009). Report of the Independent Evaluation of the Global Fund for AIDS, TB, and Malaria

(GFATM) Malaria Control Project (Round 4) - Year 3.

Graves PM, Fernando D, Attanayake N (1995) Intensified malaria control programme in Sri Lanka with

emphasis on primary care approach. Consultant report, New Health and Family Planning Project, Washington DC, IDA/World Bank.

Fernando WP, Ratnapala, PR (1988) Report on Research Project Funded by the International Science and

Technology Institute: A survey to ascertain the prevalence of G-6-PD enzyme deficiency in Sri Lanka, and its geographical and ethnical distribution. Colombo: International Science and Technological Institute.

Intercountry Consultative Meeting of National Malaria Control Programme Managers (1997-1998)

Country Report - Sri Lanka.

Kusumawathie PHD, Wickremasinghe, A.R., Karunaweera, N.D., and Wijeyaratne, M.J.S. (2005) Use of Sri

Lankan Larvivorous Fish Species in Mosquito Control. National Symposium on Mosquito Control, Programme and Proceedings: 58-69.

Lines J, Whitty, CJM, and Hanson, K. (2007) Prospects for eradication and elimination of malaria: a

technical briefing for DFID. DFID Health Resource Centre.

Office of the Director General of Health Services (2008). General Circular No. 01-14/2008: Guidelines for

Malaria Chemotherapy and the management of patients with malaria. Colombo: Director General of Health Services.

Ruberu PS (1977) Economic justification of intensive malaria control programme in Sri Lanka.

unpublished manuscript.

Samaraweera WAS (2010). Entomology Lab - Consumables and Capital Equipment from Global Fund for

AIDS, tuberculosis, and Malaria and the World Health Organization.

The World Bank (2002) Implementation Completion Report (IDA-29280) on a Credit in the Amount of

SDR 13.0 Million (U.S. $18.8 Million equivalent) to the Democratic Socialist Republic of Sri Lanka for a Health Services Project. Document of The World Bank.
